# The electrical conductivity of Al_2_O_3_ under shock-compression

**DOI:** 10.1038/srep12823

**Published:** 2015-08-04

**Authors:** Hanyu Liu, John S. Tse, W. J. Nellis

**Affiliations:** 1 Department of Physics and Engineering Physics, University of Saskatchewan, Saskatoon, S7N 5E2, Canada; 2 State key laboratory of Superhard materials, Jilin University, Changchun, 130012, China; 3 Department of Physics, Harvard University, Cambridge, MA 02138, USA

## Abstract

Sapphire (Al_2_O_3_) crystals are used below 100 GPa as anvils and windows in dynamic-compression experiments because of their transparency and high density. Above 100 GPa shock pressures, sapphire becomes opaque and electrically conducting because of shock-induced defects. Such effects prevent temperature and *dc* conductivity measurements of materials compressed quasi-isentropically. Opacities and electrical conductivities at ~100 GPa are non-equilibrium, rather than thermodynamic parameters. We have performed electronic structure calculations as a guide in predicting and interpreting shock experiments and possibly to discover a window up to ~200 GPa. Our calculations indicate shocked sapphire does not metallize by band overlap at ~300 GPa, as suggested previously by measured non-equilibrium data. Shock-compressed Al_2_O_3_ melts to a metallic liquid at ~500 GPa and 10,000 K and its conductivity increases rapidly to ~2000 Ω^−1^cm^−1^ at ~900 GPa. At these high shock temperatures and pressures sapphire is in thermal equilibrium. Calculated conductivity of Al_2_O_3_ is similar to those measured for metallic fluid H, N, O, Rb, and Cs. Despite different materials, pressures and temperatures, and compression techniques, both experimental and theoretical, conductivities of all these poor metals reach a common end state typical of strong-scattering disordered materials.

Single-crystal Al_2_O_3_, commonly called sapphire, enabled making a long-sought phase of metallic hydrogen, whose existence was predicted at the dawn of the quantum era and found experimentally as a degenerate liquid metal at high dynamic pressures above 140 GPa. The experimental conditions at which metallization was observed^1^ are consistent with Wigner and Huntington’s predicted metallization density of 9-fold compressed H-atom density in liquid H_2_ and “very low temperatures”^2^. Al_2_O_3_ was used to achieve those extreme conditions^3^, which has opened a newly accessible thermodynamic range for investigation of ultra-condensed matter. Understanding sapphire at high dynamic pressures is important for developing optical diagnostic techniques to characterize properties of unusual materials at extreme pressures and densities.

Metallic fluid hydrogen (MFH) was made in thermal equilibrium by quasi-isentropic, multiple-shock compression of liquid H_2_ contained between two disks of *c*-cut sapphire. A multiple shock wave is generated by a shock in liquid H_2_ reverberating between incompressible sapphire anvils. Measured electrical resistivities of shocked sapphire^4^ are too high to affect measurements of electrical conductivities of MFH. However, heterogeneous, non-equilibrium shock-induced defects in strong sapphire cause *c*-cut sapphire to become opaque at shock pressures in the range 100 to 130 GPa^5^, which prevents measurements of thermal emission temperatures and optical spectroscopies of MFH and other novel materials. Because of the importance of optical experiments, one of our goals is to identify transparent anvil/windows that remain transparent under dynamic pressures above 150 GPa.

Finding the solution is a challenge. Shocked sapphire anvils are out of thermal equilibrium up to some unknown shock pressure and thermally equilibrated at higher pressures. The anvils melt at some as-yet unknown high pressure and possible solid-solid transitions might occur. Relatively little is known about sapphire at shock pressures above 100 GPa; much less is known about other possible anvil materials.

To identify materials with improved properties, we are using an integrated theoretical/experimental approach. Electronic structure calculations provide insight on how to interpret experimental results and suggest possible new experiments. Electronic structure calculations yield intrinsic thermodynamic values. Opacity and electrical resistivity of sapphire at 100 GPa pressure are caused by shock-induced defects, which means they are extrinsic. Electronic structure calculations yield equilibrium intrinsic parameters that are estimates of physical limits of improvements that might be made and provide predictions of thermodynamic phenomena, such as melting. In the case of sapphire, the dynamic compression process itself can be varied. Shock-wave profiles have been measured for shocks travelling in seven directions in the sapphire rhombohedral lattice^6^, which provide indications of various degrees of shock-induced disorder. Thus, investigating effects of shock propagation in directions other than parallel to the c axis of the rhombohedral structure might lead to improved transparency in shocked sapphire.

**Giant Planets.** High pressures and temperatures occur naturally in planetary interiors, which provide challenges to understanding highly condensed matter at extreme conditions^7^,^8^. Dynamic compression achieves extreme planetary pressures and temperatures not amenable to static compression. Paradigms in this regard are measurements of electrical conductivities of synthetic Uranus (SU), a solution of water, ammonia and isopropanol representative of the Ice Giant Planets Uranus and Neptune^9^,^10^, and conductivities of liquid H_2_^1^, which comprises ~93 at.% of the Giant Planets Jupiter^7^ and Saturn and probably more than ~60 at.% of the Ice Giants^8^. All those experiments used sapphire to achieve states representative of planetary interiors. Ramp-wave compression is also quasi-isentropic and achieves thermodynamic states comparable to those achieved by multiple-shock compression^11^.

The multiple-shock technique enabled investigations of highly condensed fluid H, N and O up to 180 GPa (1.8 Mbar) and temperatures of a few 1000 K. Metallic monatomic fluid H^1^, N^12^, and O^13^ were made by band overlap on adjacent atoms at 140 GPa, 120 GPa and 100 GPa, respectively. Their metallic state is strong-scattering and its conductivity value is often called minimum metallic conductivity in which electron-scattering length is comparable to average interatomic distance. Those low-Z fluids reach a common metallic monatomic end-state at sufficiently extreme pressures and densities under quasi-isentropic compression.

Dynamic pressures in Al_2_O_3_ are determined using published Hugoniot data of Al_2_O_3_ up to 340 GPa^14^,^15^. Melting temperatures of ~6000 K for Al_2_O_3_ have been calculated at 25 GPa and 100 GPa^16^. A laser-heated diamond-anvil cell has been used to investigate the stability and compressibility of the perovskite structure of Al_2_O_3_^17^. Since sapphire (single-crystal Al_2_O_3_) is a strong material, it can have a complex two-wave elastic-plastic shock wave structure that must be taken into account in analysis of dynamic-compression experiments. For this reason, shock-wave temporal profiles have been measured, which demonstrate that above 90 GPa sapphire has only a single plastic shock wave^6^, which simplifies data analysis.

Equation-of-state Hugoniot data of Al_2_O_3_ have also been measured from 1.1 to 1.9 TPa. Those Hugoniot data of Al_2_O_3_ show that shock pressure increases rapidly with density above 500 GPa, which was proposed to be caused by a transition to a semiconducting liquid on melting^18^. The 300 K isotherm of Al_2_O_3_ has been calculated up to 370 GPa^19^. All those results are important because they demonstrate that the Hugoniot of Al_2_O_3_ has a relatively small component of thermal pressure up to ~400 GPa and thus Al_2_O_3_ is ideal for performing shock-reverberation experiments at lower dynamic pressures^3^.

Since Al_2_O_3_ is an integral part of those dynamic experiments, electrical properties of Al_2_O_3_ under dynamic compression must be determined to interpret measured conductivity voltage histories. Since metal electrodes are inserted through an Al_2_O_3_ anvil for the measurement of the electrical current and voltage of a fluid under shock reverberation^1^, it is necessary to show that the electrical resistivity of shocked Al_2_O_3_ anvils is large compared to that of a fluid sample so that current is not shunted through shocked Al_2_O_3_ rather than conducted through the fluid sample. For this reason, electrical resistivities of shocked Al_2_O_3_ were measured up to 220 GPa^4^.

At 140 GPa fluid H, N and O are strong-scattering poor metals whose conductivities are ~5 orders of magnitude higher than those of shocked sapphire (2 × 10^3^ Ω^−1^cm^−1^ for H and 10^−2^ Ω^−1^cm^−1^ for Al_2_O_3_). Thus, measured resistivities of shocked Al_2_O_3_ demonstrate negligible effect on conductivity measurements made to date. However, based on extrapolation of measured electrical conductivities of sapphire from 220 to 300 GPa, conductivity of single-shocked crystalline Al_2_O_3_ might reach that of a strong-scattering poor metal at a shock pressure of ~300 GPa^20^, which would have a major effect on analysis of conductivity data of shocked fluids above ~200 GPa. As demonstrated by electronic structure calculations herein, Al_2_O_3_ does not become a poor metal at a shock pressure of ~300 GPa. Rather, measured conductivities^20^ extrapolated to a metallic value were probably caused by shock-induced damage of the Al_2_O_3_ lattice.

A principal purpose of this paper is to report computational results on Al_2_O_3_ under high-pressure and high temperature conditions needed to qualify it for shock reverberation experiments at pressures above 180 GPa. We show that the onset of strong-scattering electrical conductivities of fluid Al_2_O_3_ occurred with a conductivity of ~200 Ω^−1^cm^−1^ at a shock pressure of ~500 GPa and 10,000 K and completes with a conductivity of 2000 Ω^−1^cm^−1^ at ~900 GPa. The substantial increase in the electrical conductivity at ~500 GPa is shown to be coincident with melting due to a substantial structural transformation from the solid to the liquid state. The densities of the melt at 10000 K and the quench solid at 300 K are 7.24 gm/cm^3^ and 7.6 gm/cm^3^, respectively. The local Al-O coordination number increased from 4-fold in the solid to 6-fold in the liquid, consequently, the band structure changed from an insulator to a metal. We have also computed the optical reflectivity of Al_2_O_3_ along the Hugoniot in Table 1 in order to estimate shock pressure at which Al_2_O_3_ is transparent to thermal radiation emitted from dynamically heated fluid to determine the temperature of a fluid sample.

A second purpose is to investigate the basic physics of a strong oxide at very high shock pressures. First, to provide scientific information on how breaking strong (~5 eV) chemical bonds of Al_2_O_3_ affects structural and electrical properties of strong oxides at high shock pressures and temperatures^21^, and to answer the question as to whether a strong weakly-compressible oxide might also reach a metallic end-state at sufficiently extreme pressures and temperatures, similar to the one observed for compressible liquids H_2_, N_2_ and O_2_ as they crossover to monatomic, strong-scattering metallic H, N and O. Because of strong bonds in Al_2_O_3_ such a cross over is expected at much higher shock pressures than ~100 GPa as for H, N and O^1^,^12^,^13^.

## Results and Discussions

To explore the physical, electrical and optical properties of Al_2_O_3_, we have computed the equation of state (EOS) of Al_2_O_3_. A crucial measure for theoretical EOS data is the principle Hugoniot curve, describing the shock adiabatically through a relation between the initial and final internal energy, pressure and volume, respectively, (*E*_0_, *P*_0_, *V*_0_) and (*E*_1_, *P*_1_, *V*_1_) according to the following Rankine-Hugoniot equation:


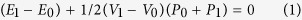


where the internal energy *E*_*1*_ at temperature *T*_*1*_ is the sum of the ion kinetic energy 3/2*k*_*B*_*T*_*1*_, the time average of the DFT potential energy, and zero-point energy with *k*_*B*_ being the Boltzmann constant. The pressure consists of contributions from the electronic *P*_*e*_ and ionic *P*_i_ components. The electronic pressure part is evaluated using the force provided by first-principle simulations, which contains contributions from the direct ion-ion interaction and a term from the gradient of the electronic total energy. The ionic pressure part is computed from the ideal gas expression *P* = *P*_*e*_ + *ρ*_*n*_*k*_*B*_*T*, where *ρ*_n_ is the number density. For the sapphire principal Hugoniot curve, the initial density is *ρ* = 4.0 g/cm^3^.

**Figure 1:**
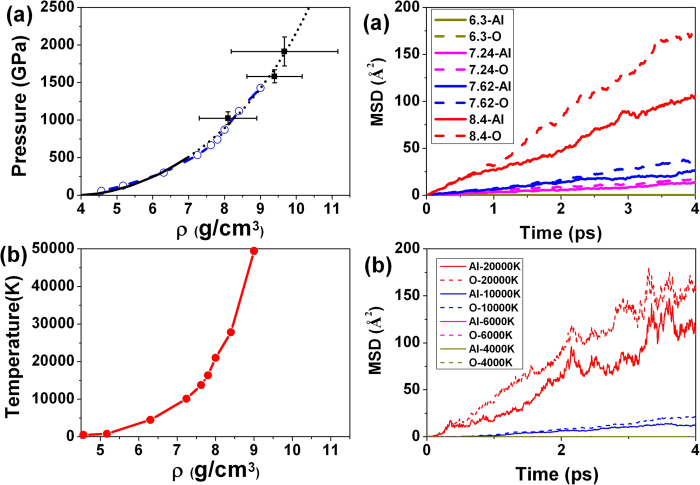
Hugoniot equation of state for shocked Al_2_O_3_. Calculated (**a**) pressure dependence with density (circles symbols) with experimental data (continuous lines and squares with error bars) taken from Refs 13, 17. (**b**) Calculated temperature dependence with density (solid symbols).

The Hugoniot points are determined by the following way. For a given density, a fixed supercell of 32 Al and 48 O atoms was constructed from the ambient-pressure corundum structure. A series of *ab initio* molecular dynamics simulations were performed for different temperatures *T*. For a given density and a set of temperatures, we plotted (*E*_1_ − *E*_0_) along with 1/2(*V*_1_ − *V*_0_)(*P*_0_ + *P*_1_) as a function of temperature. The intersection of the two curves fixed the principal Hugoniot point (*E*_1_, *P*_1_, *V*_1_) that satisfies Eq. (1). The Hugoniot curve and the relevant optical and electrical data for Al_2_O_3_ are reported in Fig. 1 and Table 1. It is seen that the theoretical results are in agreement with experimental result. At *ρ* = 9.0 g/cm^3^, the calculated pressure is 1429 GPa and temperature is 49,457 K. The good agreement between the theoretical and the experimental Hugoniot validates the parameters and methodology chosen for the calculations. However, it is important to point out that a measured Hugoniot is relatively insensitive to disorder of a material and defects were not included in the calculations. Thus, this comparison is essentially a self-consistency check. All the calculations were performed under hydrostatic conditions with the pressure and temperature increased in steps.[Table t1]

The electrical conductivity is calculated using the most general formulation given by the Kubo-Greenwood (KG) formulation without assumptions made on the ionic structure or on the electron-ion interactions. This method did not include the electron-electron scatterings. In the framework of the independent particle approximation, the Kubo-Greenwood formulation gives the real part of the electrical conductivity as a function of frequency *ω*,


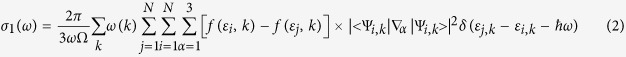


where *ω* is the frequency, Ω is the volume of the supercell, *N* is the total number of energy bands used, Ψ_*i,k*_ and ε_*i,k*_ are the electronic eigenstates and eigenvalues for the electronic state *i* at *k*, *f*(ε_*i,k*_) is the Fermi distribution function, and *ω*(*k*) represents the *k*-point weighing factor. Other relevant properties can be derived from the frequency-dependent real part of the electrical conductivity. It is noteworthy that this equation does not fully take into account electron-electron scattering effects.

**Figure 2:**
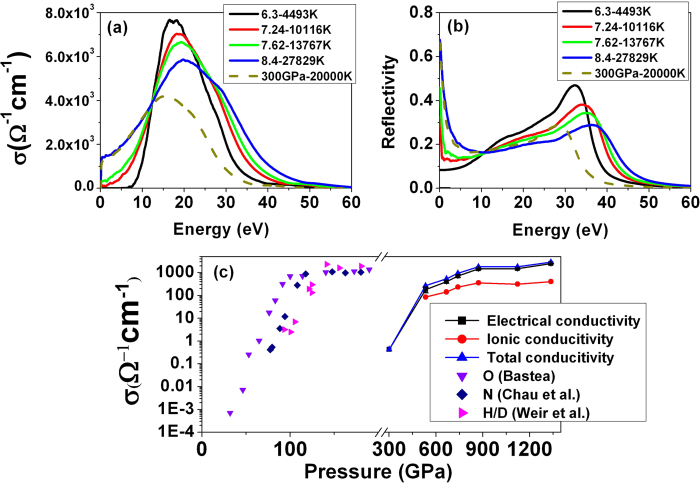
The calculated electrical conductivity of Al_2_O_3_. (**a**) The real part of dynamic electrical conductivity along the principal Hugoniot. The results were obtained from the average over 10 uncorrelated MD configurations. (**b**) the calculated reflectivity along the principal Hugoniot. (**c**) Calculated electrical conductivitives along the principal Hugoniot of Al_2_O_3_ are shown on the right. For comparison, the measured conductivities of N^12^, H^7^ and O^13^ from multiple shock experiments are shown on the left. The dc conductivity *σ*_*dc*_ can be estimated from the frequency dependence *σ*(*ω*) (**a**) in the limit at which *ω* → 0.

The behavior of the frequency-dependent conductivity and reflectivity at different Hugoniot points is shown in Fig. 2. It is found that the conductivity at four different densities exhibit a common peak around 20 eV. This can be associated with transitions to the lowest excited states. With increasing density and temperature along the principal Hugoniot, the shape of the conductivity remains the same, but the main peak shifts to lower frequencies, and eventually led to a significant increase in dc conductivity. The *dc* conductivity *σ*_*dc*_ can be estimated from the frequency dependence *σ*(*ω*) in the limit at which *ω* → 0. Significant variation of the *dc* conductivity along the principal Hugoniot is depicted in Fig. 2c. The highest measured *dc* conductivity of sapphire below 300 GPa is 5 Ω^−1^cm^−1^ at 220 GPa^4^ The low *dc* conductivity is simply due to the fact that Al_2_O_3_ is still in the insulating state. Above 500 GPa, the calculated *dc* conductivity increases rapidly and reaches 181 Ω^−1^cm^−1^ at 533 GPa. At 1112 GPa, the *dc* conductivity attains a value of 1515 Ω^−1^cm^−1^. This value is typical of strong-scattering metallic fluids H, N and O at 100 GPa pressures^1^,^12^,^13^.

It is well known that energy dissipation from shock compression is converted into temperature *T* and entropy *S*. The relative contributions of the two are determined by the nature of chemical bonding in the material. The extremes are compressible van der Waals fluids and weakly compressible strong materials, such as oxides. In this case, up to a shock pressure of ~900 GPa, the pressure is caused by the compression of the relatively strong Al_2_O_3_ lattice and a modest shock-induced thermal pressure of the electrons. In this regime, irreversible shock energy is mainly converted to compressing, disordering and heating of the Al_2_O_3_ lattice and from the heating and excitation of electrons across the mobility gap into the conduction band of disordered Al_2_O_3_. At ~900 GPa, the shock-induced entropy is essentially saturated and shock energy at higher pressures transfers preferentially into thermal energy. Above 900 GPa, the electrical conductivity essentially plateaus at ~2000 Ω^−1^cm^−1^. This trend is typical of strong-scattering fluid metals such as H, N and O which have a similar conductivity. The present results show strong solid oxides at high dynamic pressures approaching ~TPa have an electrical conductivity similar those of compressible fluid metals. The essential difference is that H, N and O reach the maximum conductivity at 100–140 GPa at 3000 K or higher. In comparison, to reach the maximum conductivity, strong Al_2_O_3_ and, probably other oxides, requires dynamic pressures up to 900 GPa or even higher and shock-induced temperatures of ~30,000 K. The details are dependent on oxide bond strength and shock-induced thermal energy.

**Figure 3:**
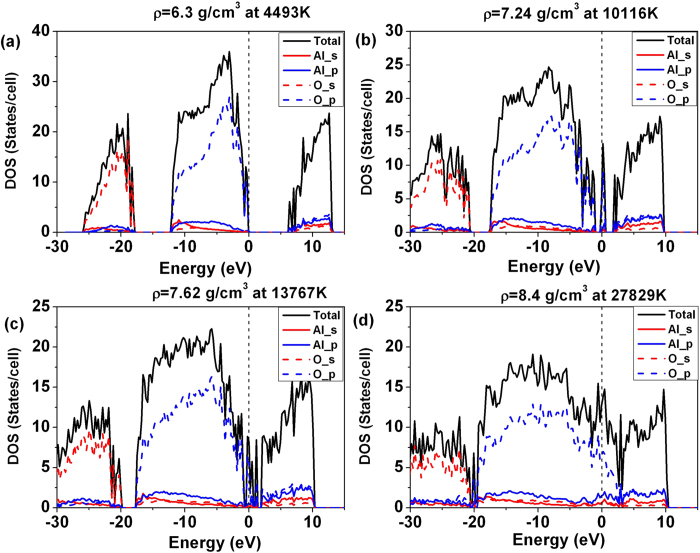
Total and projected density of states of Al_2_O_3_ along the Hugoniot. Vertical dashed line indicates the Fermi level. The calculated DOSs support the conductivity calculations. The DOS shows the 2*p* orbital of O and 3*p* orbital of Al is responsible for the electron conductivity.

To explain the trend in the *dc* conductivity, we have computed the density of states (DOS) of Al_2_O_3_ along the Hugoniot (Fig. 3). The results support the conductivity calculations. In the calculated DOS the occupied states at low density clearly split into the O-2*s* band (−26 eV to −18 eV) and the O-2*p* band (−11 eV to 0 eV). The band gap energy separating the occupied states at the top of the valence band and the conduction band decreases with increasing pressure. At *ρ* = 6.3 g/cm^3^, the band gap was calculated to be approximately 6 eV. At *ρ* = 7.24 g/cm^3^, localized mid-gap band of predominately O-2*p* character starts to emerge. The increased conductivity depends on the coupled closure of the energy gap. The DOS shows the 2*p* orbital of O and 3*p* orbital of Al are responsible for the electron conductivity. It is noteworthy that sapphire becomes opaque and electrically conducting because of shock-induced defects above 100 GPa shock pressures. The investigation of shock-induced effects will require substantially larger supercell and this is beyond the scope of this study.

**Figure 4:**
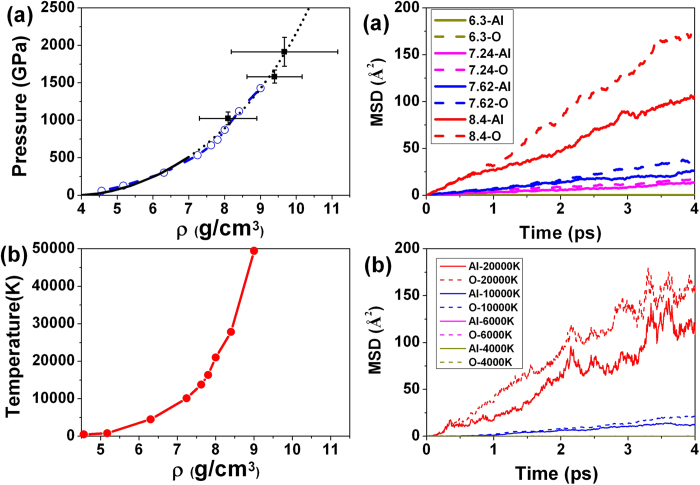
Mean squared displacements (MSD) of Al_2_O_3_. (**a**) MSD of Al and O ions in corundum-Al_2_O_3_ structure along the Hugoniot. The numerical value preceding the atomic symbol is the density (g/cm^3^). This indicates melting occurs by a crossover in a range of densities between 7.24 and 8.4 g/cm^3^. (**b**) Mean squared displacements of Al and O ions of perovskite-Al_2_O_3_ at 300 GPa and different temperatures.

The atomic mean square displacement (MSD) is an important parameter to investigate the diffusion of a system. The MSD can be calculated for the atomic displacements as a function of time, 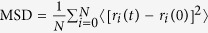, where *N* is the total number of total atoms in the system, *r*_*i*_(*t*) is the atomic position at a time of t and *r*_*i*_(0) is the initial position of atom *i*, and <…> represents the ensemble average. We have computed the MSD of Al and O atoms for Al_2_O_3_ in MD simulations along the Hugoniot. The results summarized in Fig. 4a show unambiguously that Al_2_O_3_ is a liquid with mobile Al and O atoms at *ρ* = 7.24 g/cm^3^ and 533 GPa. Incidentally this corresponds to the pressure of 533 GPa where the *dc* conductivity has increased. From the MSD (Fig. 4a), the diffusion coefficients of Al and O atoms are calculated to be 3.24 × 10^−4^ cm^2^/s and 4.24 × 10^−4^ cm^2^/s at 10,116 K and *ρ* = 7.24 g/cm^3^, 6.69 × 10^−4^ cm^2^/s and 9.57 × 10^−4^ cm^2^/s at 13,767 K and *ρ* = 7.62 g/cm^3^ and 2.657 × 10^−3^ cm^2^/s and 4.306 × 10^−3^ cm^2^/s at 27,829 K and *ρ* = 8.4 g/cm^3^, respectively. The ionic conductivity can be estimated from the diffusion rate making use of the Nernst-Einstein relationship: *σ* *=* *n*(*ze*)^2^*D*/*k*_*b*_*T*, where *σ* is the conductivity, *D* is the diffusion coefficient, *n* represents the ionic concentration (*i.e.,* the number of ions per unit volume), *z* is the valence of the charge carrier (in this case, *z*_*Al*_ = +3 and *z*_*O*_ = −2), *e* the elementary charge of an electron, *k*_*b*_ is the Boltzmann constant and *T* the temperature. The calculated bulk ionic conductivity value is 85.9 Ω^−1^cm^−1^ at 10,116 K and *ρ* = 7.24 g/cm^3^, 142.7 Ω^−1^cm^−1^ at 13,767 K and *ρ* = 7.62 g/cm^3^ and 313 Ω^−1^cm^−1^ at 27,829 K and *ρ* = 8.4 g/cm^3^, respectively. Note that the total conductivity is a sum of the electrical and ionic contributions. The calculated *dc* conductivity as a function of pressure is shown in Fig. 2c. At low pressures (e.g. < 500 GPa), the ionic conductivity plays an important role on the overall the electrical conductivity. Above 500 GPa, the electrical conductivity dominates significantly.

**Figure 5:**
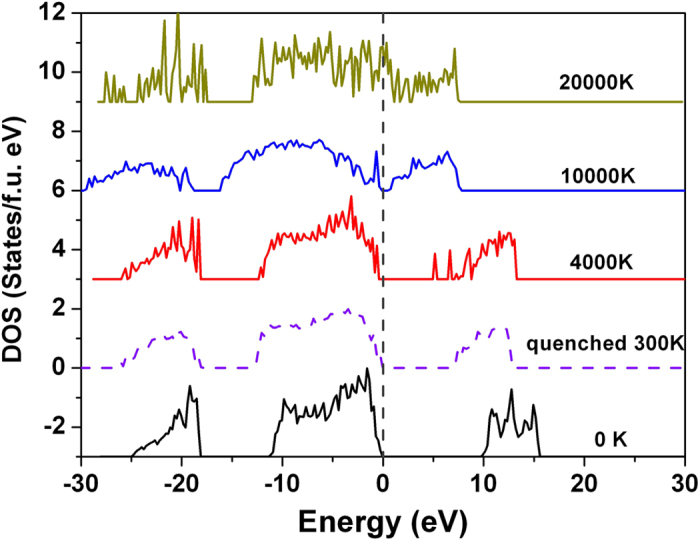
Total density of states of perovskite-Al_2_O_3_ at 300 GPa and different temperatures. The vertical dashed line indicates the Fermi level. This phase has a band gap of 10 eV at 300 GPa and 0 K. With increasing temperatures, the band gap becomes smaller due to the lowering of the conduction bands. At 10,000 K, the band gap of Al_2_O_3_ is closed.

For completeness, we have also studied the melt obtained from the perovskite-Al_2_O_3_ structure which was reported to be stable above 130 GPa^17^,^22^. As shown in Fig. 5, the solid phase has a band gap of 10 eV at 300 GPa and 0 K. With increasing temperatures, the band gap becomes smaller. Similar to the corundum phase, at 10,000 K, the Al_2_O_3_ has melted and the gap closed as shown in Figs 4 and 5. It is clear that the band gap closure indicated the enhanced metallic behavior derives from the excitation to the lower conduction bands (Fig. 5). The main peak in the conductivity is located at around 16 eV. The *dc* conductivity *σ*_*dc*_ estimated from 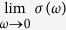 is ~1000 Ω^−1^cm^−1^. We then quenched the high-temperature structure to room temperature at 300 GPa. The quenched glass became an insulator with a band gap of 7 eV (Fig. 5).

We have also investigated the MSD for the dynamical behavior of the Al and O atoms of the quenched solid at 300 GPa. Below 6000 K, the Al and O atoms are not diffusive. Above 10,000 K the Al and O atoms become mobile and Al_2_O_3_ is a liquid (Fig. 4b). Once again, the solid to liquid transition coincides with metallization. The diffusion coefficients of Al and O atoms are calculated from the MSD are found to be 5.7 × 10^−4^ cm^2^/s and 9.5 × 10^−4^ cm^2^/s at 10,000 K and 300 GPa and 2.5 × 10^−3^ cm^2^/s and 3.6 × 10^−3^ cm^2^/s at 20,000 K and 300 GPa. The diffusion rate is one order of magnitude larger at 20,000 K as compared to 10,000 K. The calculated ionic conductivity of the melt from Nernst-Einstein equation is 144 Ω^−1^cm^−1^ at 10,000 K and 257 Ω^−1^cm^−1^ at 20,000 K and at 300 GPa. Therefore, the total conductivity is 1746 Ω^−1^cm^−1^ at 300 GPa and 20,000 K. It is important to point out that no substantial difference was found in the physical and electronic properties of the liquids obtained by melting the corundum (*vide supra*) and perovskite phases of Al_2_O_3_.

Finally, we wish to comment on the calculations. We have tested the system size effect on the electrical conductivity by repeating the calculation on melted Al_2_O_3_ at 6.3 g/cm^3^ using a larger supercell consisted of 270 atoms. The result is shown in Fig. S1 and no significant difference with that obtained with a smaller supercell was found. Furthermore, we made no attempt to accurately determine the melting curve of Al_2_O_3_. The molten states were generated by heating the structure in the MD box by raising the temperature. In the absence of a liquid-solid interface, the “melting” condition should correspond to mechanical melting which has a temperature higher than thermodynamic melting^23^. The effect of electron-electron scattering on the electrical conductivity has been neglected. Under the pressure and temperature conditions studied here, we expect Al_2_O_3_ is still degenerate and electron-electron scatterings may only increase the electrical conductivity slightly but will not alter the predicted temperature trend significantly. On the other hand, the importance of electron-electron scattering processes to the electrical conductivity is still a subject of debate. A recent calculation on the electrical conductivity of Fe at high temperature and high pressure^24^ shows the effects are important. This observation is contradicted by another study^25^, which shows the opposite result showing Fe is still a Fermi liquid even at high pressure-temperature conditions. We will not elaborate this controversy further.

## Conclusion

*Ab initio* molecular dynamics simulations for Al_2_O_3_ under shocked compression up to ~1500 GPa have been performed. Finite electrical conductivity in Al_2_O_3_ is predicted to exist only in the melt, as the quenched melt is shown to be an insulator. Al_2_O_3_ conductivity at 900 GPa of 2000 Ω^−1^-cm^−1^ is typical of the conductivities of metallic fluid H, N, and O on completion of crossovers from diatomic insulators to strong-scattering monatomic fluid metals under quasi-isentropic compression. The simulated EOS of Al_2_O_3_ is in good agreement with experimental data for the principal Hugoniot. The *dc* conductivity was found to increase rapidly above 500 GPa, In addition, the calculated mean squared displacements suggest Al_2_O_3_ is liquid above ~10,000 K. The present results provide an avenue to understanding of the metallization for shocked Al_2_O_3_.

### Computational details

*Ab initio* MD simulations for Al_2_O_3_ were performed with the *NPT* (*N*-number of particles, *P*-pressure, *T*-temperature) ensemble recently implemented in *Vienna Ab initio Simulation Package* (VASP) code^26^,^27^,^28^. The all-electron projector-augmented wave^29^ (PAW) method was used. Exchange and correlation effects were treated in generalized-gradient approximation (GGA)^30^. We have employed simulation models containing 80 atoms (32 Al atoms and 48 O atoms) for Al_2_O_3_. The duration of the simulation was at least 5 ps. A plane-wave cutoff of 500 eV was used. Brillouin zone sampling of 3 × 3 × 3 *k*-meshes for the supercell was employed. The time step used in the MD simulation was 1 fs and the self-consistency on the total energy was chosen to be 1 × 10^−5^ eV.

## Additional Information

**How to cite this article**: Liu, H. *et al.* The electrical conductivity of Al_2_O_3_ under shock-compression. *Sci. Rep.* **5**, 12823; doi: 10.1038/srep12823 (2015).

## Supplementary Material

Supplementary information

## Figures and Tables

**Table 1 t1:** Temperature, pressure and conductivity, reflectivity of Al_2_O_3_ along the Hugoniot.

ρ	T	P	σ	σ_r_	σ_g_	σ_b_	σ_db_	κ_r_	κ_g_	κ_b_	κ_db_
4.56	500	57	0	0	0	0	0	0	0	0	0
5.17	796	126	0	0	0	0	0	0	0	0	0
6.3	4493	300	0.43	0.8	1.3	1.7	3.1	0.09	0.09	0.09	0.09
7.24	10116	533	266	185	238	224	303	0.13	0.13	0.12	0.12
7.62	13767	667	542	432	501	523	663	0.17	0.16	0.15	0.15
7.8	16372	740	949	544	563	629	769	0.18	0.17	0.16	0.16
8	21006	872	1827	1614	1568	1606	1712	0.33	0.28	0.26	0.23
8.4	27829	1112	1828	1501	1527	1570	1711	0.32	0.28	0.26	0.23
9	49457	1429	2904	2561	2585	2621	2757	0.43	0.39	0.36	0.33

σ_r_, σ_g_, σ_b_, σ_db_, κ_r_, κ_g,_ κ_b_ and κ_db_, indicate conductivity (σ) and reflectivity (κ) at red (633 nm), green (508 nm), blue (442 nm) and deep blue laser (350 nm), where subscripts r, g, b and db mean red, green, blue and deep blue, respectively. The respective units for density, temperature, pressure and conductivity are g/cm^3^, K, GPa and Ω^−1^cm^−1^.
