# Supplementary material for: The electrical conductivity of Al2O3 under shock-compression
Source: Sci Rep. 2015 Aug 4;5:12823. doi: 10.1038/srep12823 (PMC4523845; doi:10.1038/srep12823)
Supplement: Supplementary information [file srep12823-s1.pdf]

Supplementary materials for

The electrical conductivity of Al_2O_3 under shock-compression

Hanyu Liu[1], John S. Tse[1,2*], W. J. Nellis[3]

[1]Department of Physics and Engineering Physics, University of Saskatchewan, Saskatoon, Canada, S7N 5E2

[2]State key laboratory of Superhard materials, Jilin University, Changchun, China 130012

[3]Department of Physics, Harvard University, Cambridge, USA, MA 02138

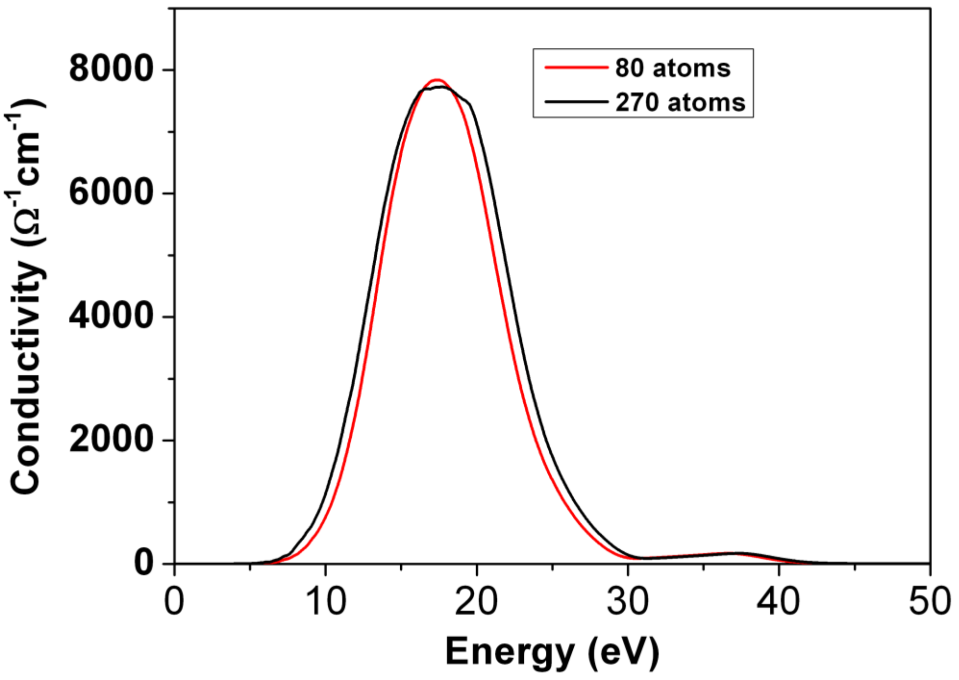

Fig. S1 The calculated conductivity of Al_2O_3 at density of 6.3 g/cm^3 using a supercell consisted of 80 and 270 atoms. The results indicate there is no significant change using larger supercell, suggesting the simulation with 80 atoms is good enough to study the electronic properties of Al_2O_3 at high pressure.

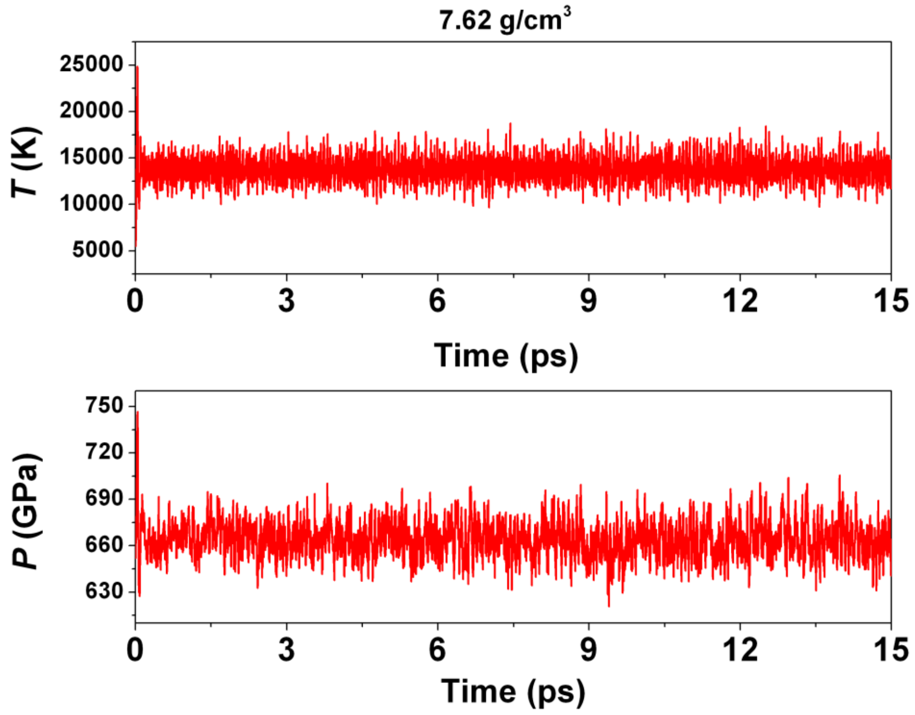

Fig. S2 The temperature and pressure were shown as a functional of simulated time at 7.62 g/cm^3. It indicates the system can reach equilibrium after several ps simulations.
